# CATCH-UP vaccines: protocol for a randomized controlled trial using the multiphase optimization strategy (MOST) framework to evaluate education interventions to increase COVID-19 vaccine uptake in Oklahoma

**DOI:** 10.1186/s12889-023-16077-w

**Published:** 2023-06-14

**Authors:** Amanda E. Janitz, Jordan M. Neil, Laura A. Bray, Lori L. Jervis, Laura Ross, Janis E. Campbell, Mark P. Doescher, Paul G. Spicer, Mary L. Williams, April K. Lopez, Conce A. Uribe-Frias, Sixia Chen, Judith A. James, Timothy M. VanWagoner

**Affiliations:** 1 University of Oklahoma Health Sciences Center, 801 NE 13th St., CHB 309, Oklahoma City, OK 73104 USA; 2 University of Oklahoma Norman Campus, 5 Partners Place, 201 Stephenson Parkway, Suite 4100, Norman, OK 73072 USA; 3 Public Health Institute of Oklahoma, P.O. Box 60926, Oklahoma City, OK 73146 USA; 4 Oklahoma Medical Research Foundation, 825 NE 13th Street, Oklahoma City, OK 73104 USA

**Keywords:** Multiphase optimization strategy, COVID-19, Vaccine hesitancy, Underserved communities, Rural health disparities, Community-engaged intervention

## Abstract

**Background:**

Oklahoma’s cumulative COVID-19 incidence is higher in rural than urban counties and higher than the overall US incidence. Furthermore, fewer Oklahomans have received at least one COVID-19 vaccine compared to the US average. Our goal is to conduct a randomized controlled trial using the multiphase optimization strategy (MOST) to test multiple educational interventions to improve uptake of COVID-19 vaccination among underserved populations in Oklahoma.

**Methods:**

Our study uses the preparation and optimization phases of the MOST framework. We conduct focus groups among community partners and community members previously involved in hosting COVID-19 testing events to inform intervention design (preparation). In a randomized clinical trial, we test three interventions to improve vaccination uptake: (1) process improvement (text messages); (2) barrier elicitation and reduction (electronic survey with tailored questions/prompts); and (2) teachable moment messaging (motivational interviewing) in a three-factor fully crossed factorial design (optimization).

**Discussion:**

Because of Oklahoma’s higher COVID-19 impact and lower vaccine uptake, identifying community-driven interventions is critical to address vaccine hesitancy. The MOST framework provides an innovative and timely opportunity to efficiently evaluate multiple educational interventions in a single study.

**Trial Registration:**

ClinicalTrials.gov: NCT05236270, First Posted: February 11, 2022, Last Update Posted: August 31, 2022.

## Background

As of February 2023, 1,281,551 cases of COVID-19 and 17,827 deaths have occurred in Oklahoma [1]. Oklahoma’s cumulative mortality is higher in rural (430 per 100,000) compared to urban (320 per 100,000) counties [2]. This is particularly problematic due to the current state of rural healthcare including the lack of providers and recent closure of many rural hospitals in Oklahoma [3]. Even before the pandemic, rural healthcare providers [4] and hospitals were under stress that threatened care delivery in these regions [5]. Rural providers and hospitals are often under-resourced; and the existing health disparities in rural America that exacerbate COVID-19 cases may further strain capacity in these settings [6, 7]. Oklahoma is a highly rural state with 1.3 million residents (33%) living in rural areas in 2021, more than twice the national average of 14% [8, 9]. High rates of tobacco use, obesity, poor health literacy, and other health risk factors, often compounded by the additional distance required to access care and an older population, contribute to the high incidence and mortality rates for a range of chronic conditions among underserved rural populations [10].

**Fig. 1:**
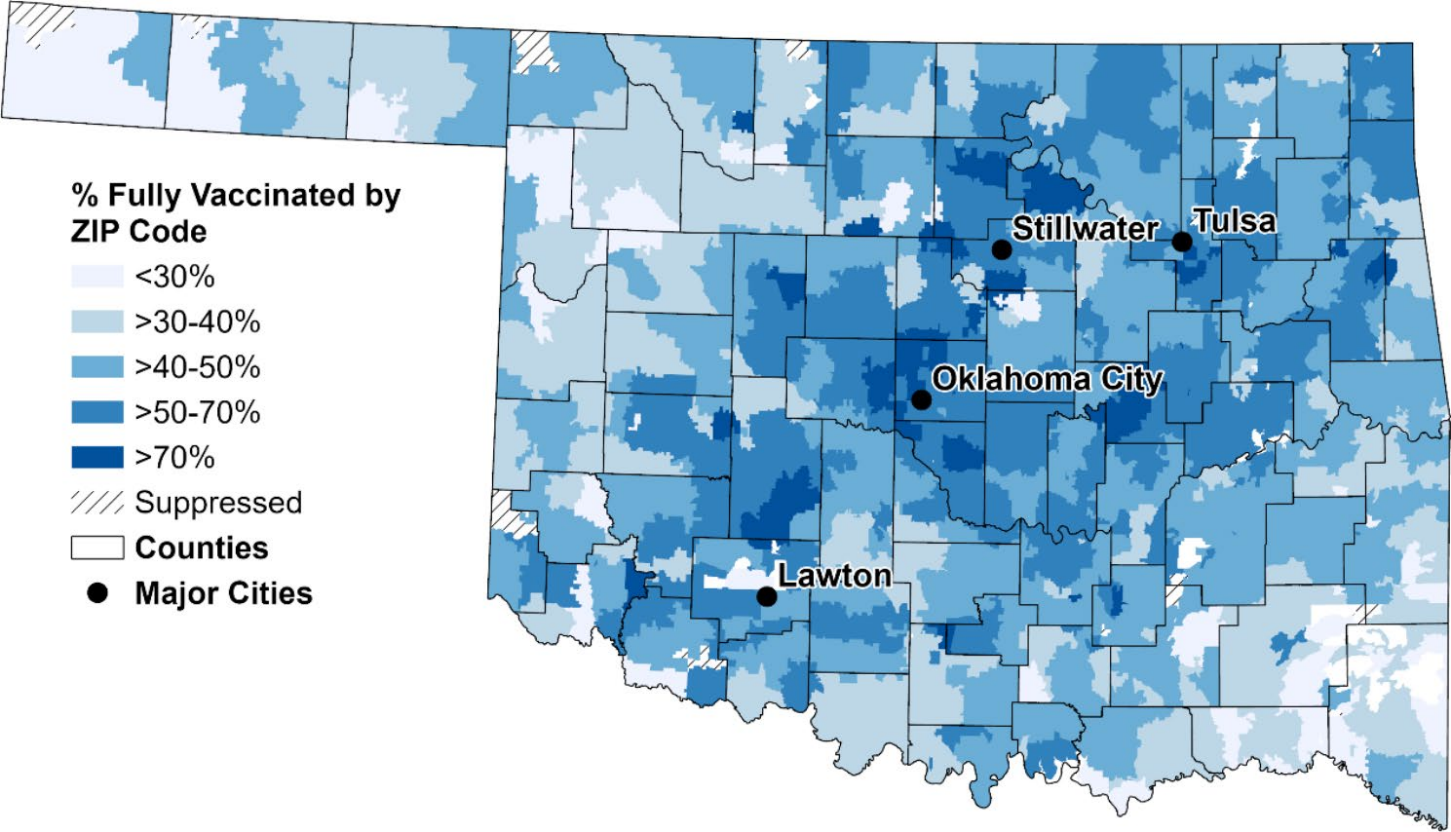
COVID-19 vaccine uptake (completion of the primary vaccine series) by ZIP Code in Oklahoma as of February 8, 2023

Over 6.7 million COVID-19 vaccines have been administered in Oklahoma, with 75% of the population having received one dose, 60% fully vaccinated with the primary vaccine series, and 11% with an updated bivalent booster [2]. Vaccine uptake is particularly low in the southeastern and northwestern areas of the state (Fig. 1). The most common reason for vaccine hesitancy when initially developing our project in April 2021 was concern about vaccine side effects and safety (30%) [11]. Other reasons for hesitation included waiting for more data/information (15%), lack of trust in government (13%), and concerns about how rapidly vaccines have been developed (10%) [11].

The COVID-19 pandemic has presented unique challenges to health communication and messaging, with limited knowledge about what interventions are most effective in increasing COVID-19 vaccine uptake. Studies have indicated that tailored, community-engaged approaches are necessary to address vaccine hesitancy, particularly in rural and underserved communities [12–23]. This emphasizes the need for studies to evaluate tailored, community-based interventions through multiple modalities, including text messages, written educational messaging, and personal interactions with community members to determine the most effective strategies for reducing vaccine hesitancy and increasing vaccine uptake.

Conceptually rooted in engineering, MOST (multiphase optimization strategy) emphasizes incremental, evidence-based advancement in intervention science to ensure optimal COVID-19 vaccine uptake in underserved populations. The goal of MOST is to identify interventions that are effective, economical, efficient, and scalable [24]. To understand which interventions are most effective, we use MOST to address the following research aims: (1) Identify COVID-19 vaccination barriers/facilitators and assess acceptability and feasibility of a suite of evidence-based vaccine intervention strategies among Oklahoma’s rural, minority, and high-risk populations to inform a targeted multicomponent intervention; and (2) Develop and optimize a multicomponent intervention to improve COVID-19 vaccination among Oklahoman’s seeking COVID-19 testing events.

## Methods/Design

### Overview

This project is an extension of a COVID-19 testing project, which included both viral and antibody point-of-care testing, funded through the Rapid Acceleration of Diagnostics – Underserved Populations (RADx-UP) initiative through the National Institutes of Health (NIH) [25]. CATCH-UP (Community - engaged Approaches to Testing in Community and Healthcare Settings for Underserved Populations) Vaccines has a goal of addressing vaccine hesitancy in Oklahoma and was funded as a supplement to the CATCH-UP grant (which is a supplement to the Oklahoma Shared Clinical and Translational Resources parent grant). The parent CATCH-UP project works closely with our community partner, Public Health Institute of Oklahoma (PHIO), to identify partners interested in providing COVID-19 testing in their communities either through hosting dedicated events or sponsoring booths at broader events. Most of the CATCH-UP events prior to 2022 offered viral polymerase chain reaction (PCR)-based and/or rapid antigen-based testing. Some events also included serology testing by lateral-flow assays (LFA), which proved to be highly popular and was a major driving force for community acceptance of the CATCH-UP events. While LFA tests are not recommended for clinical-decision making, the CATCH-UP researchers observed that they did provide a convenient entry-point for discussions about the need for vaccination, particularly among individuals who had either documented cases of COVID-19 or believed they may have contracted the virus early in the pandemic and were undecided about vaccination. Due to these factors, our team decided to develop our vaccine hesitancy intervention around testing events and build on the program implemented through CATCH-UP.

### Methodologic Framework

The proposed study employs MOST, which is a methodological approach for building, optimizing, and evaluating multicomponent interventions [24, 26]. Our study uses the preparation and optimization phases of the MOST framework. In the preparation phase, we conduct focus groups among volunteers as described above. In the optimization phase, we test three interventions to improve vaccination uptake: (1) process improvement (text messages); (2) barrier elicitation and reduction (electronic survey with tailored questions/prompts); and (3) teachable moment messaging (motivational interviewing).

### Aim 1 (Preparation)

Identify COVID-19 vaccination barriers/facilitators and assess acceptability and feasibility of a suite of evidence-based vaccine intervention strategies among Oklahoma’s rural, minority, and high-risk populations to inform a targeted multicomponent intervention.

### Study Design

We are conducting up to 10 focus groups including participants from communities with low levels of vaccine uptake and/or high levels of vaccine hesitancy and communities holding events where COVID-19 testing or vaccination is offered in partnership with the CATCH-UP program. We recruit focus group participants in conjunction with testing events and through conversations with community leaders prior to testing events. Focus groups: (1) explore vaccine-related concerns and motivators, (2) evaluate educational material for local and cultural acceptability; and (3) examine outreach and communication intervention strategies detailed in the NIH COVID-19 Vaccination Communication white paper [27] for feasibility to be implemented at later testing events.

### Setting and Focus Group Procedure

Focus groups are conducted both virtually (via teleconferencing/telephone) or in-person. The focus group guide was developed in conjunction with our collaborative team. Focus groups examine barriers and facilitators to vaccination within each community (e.g., perceptions of COVID-19 severity, vaccine concerns, vaccine availability/accessibility, competing priorities, etc.). Educational material is examined for local and cultural acceptability. Participants are presented with several vaccine interventions selected by the team for demonstrated efficacy as well as likely acceptability among Oklahoma communities. Responses to selected interventions are elicited, with special attention to perceived effectiveness and acceptability within their respective communities, barriers to/facilitators of implementation, and needed modifications for local/cultural relevance. We ask participants to complete a brief demographic survey that is de-identified. We expect to enroll a maximum of 60 focus group participants (approximately 6 participants per focus group).

### Inclusion criteria

To be eligible for focus group participation, participants must be 18 years of age or older, have a leadership/membership role in community organization or staff at CATCH-UP testing event, and speak English.

### Recruitment

Focus groups include community leaders and staff/volunteers involved in community events, primarily COVID-19 testing events in collaboration with PHIO. Included participants are able to provide insights into the barriers faced within their communities in Oklahoma in partnership with the CATCH-UP program. We recruit focus group participants through emails and advertisements to the community organizations. We advertise for the focus groups through social media, websites, email, and flyers. Testing event partners disseminate flyers during community team meetings and at CATCH-UP testing events and in partnership with PHIO. At the time of contact, we explain the purpose of the project and the nature of the study. Participants receive a $40 gift card as compensation for their participation. We expect the focus groups to last about one hour.

### Analysis

Digitally recorded focus groups are securely uploaded to protected servers and transcribed using NUDIST Vivo Transcription. Transcriptions are team coded for conceptual themes using QSR NUDIST Vivo12 Pro for Windows [28]. Throughout the coding process, qualitative data are categorized, connections made between categories, and core categories identified and systematically linked to other categories. Focus group data inform intervention selection and needed modifications for community acceptability/relevance.

### Aim 2 (optimization)

Develop and optimize a multicomponent intervention to improve COVID-19 vaccination among Oklahoman’s seeking COVID-19 testing at CATCH-UP testing events.

### Overall Study Design

The purpose of this intervention study is to increase intention to receive a COVID-19 vaccine among community members who are eligible to receive at least one dose. To do so, this randomized controlled trial (n = 400) employs a factorial design to assess three intervention components to improve vaccine uptake that vary on: (1) process improvement (text messages); (2) barrier elicitation and reduction (electronic survey with tailored questions/prompts); and (3) teachable moment messaging (motivational interviewing) (Tables 1 and 2).

**Table 1:** Experimental conditions in the 2^3^ factorial design for the COVID-19 vaccine intervention

	Factor		
Condition	Process Improvement	Barrier Elicitation and Reduction	Teachable Moment Messaging
1	No	No	No
2	No	Yes	No
3	No	No	Yes
4	No	Yes	Yes
5	Yes	Yes	Yes
6	Yes	No	Yes
7	Yes	No	No
8	Yes	Yes	No

**Table 2:** Intervention factors being evaluated in the study

	Description	
Factor	Experimental	Control
Process Improvement	Text message encouraging vaccine uptake	No text message
Barrier Elicitation and Reduction	Tablet-based tailored educational messaging about vaccines with barriers addressed	Attention control educational message (health and wellness)
Teachable Moment Messaging	Motivational interviewing focused on antibody test interpretation and encouragement of vaccine uptake using 5 A’s (Ask, Advise, Assess, Assist, Arrange)	Interpretation of antibody results and encouragement of vaccine uptake

Our primary outcome is intention to receive a COVID-19 vaccine immediately after the intervention. We measure this with the question “How likely are you to get an approved COVID-19 vaccine?” and “If you have received 1 dose of the Janssen vaccine or 2 doses of the Moderna or Pfizer vaccine, how likely are you to get an approved booster shot?” Response options are ordinal ranging from “very likely” to “definitely not”, with additional options for “don’t know”, “prefer not to answer”, and “not applicable (I’ve already received a COVID-19 vaccine/booster)”. Our secondary outcome is vaccine uptake measured 30 and 60 days after the study interventions are complete. We obtain this information through self-report of receiving a dose of any approved vaccine after the intervention is complete.

### Setting

The study sites are determined by our community partner, PHIO, based on an application process coordinated by PHIO. Our goal is to identify intervention sites in each quadrant of the state, as well as one site in the Oklahoma City metro area (central Oklahoma), and one site in the Tulsa metro area (northeast Oklahoma). Potential sites include churches, hospitals, schools, and community settings (e.g., park, local business) in both rural and urban locations. Further sites will be considered for study inclusion in accordance with the wishes of our community partners and gaps identified in participation. We are using Research Electronic Data Capture (REDCap) software (Nashville, TN) for all study activities. Approved research staff enroll participants and conduct interventions through their university REDCap account.

### Inclusion criteria

To be eligible for intervention participation, participants must be 18 years of age or older, eligible to receive a COVID-19 vaccine dose at the time of consent based on current Centers for Disease Control and Prevention (CDC) guidelines [29], and able to read and speak English.

### Recruitment

**Fig. 2:**
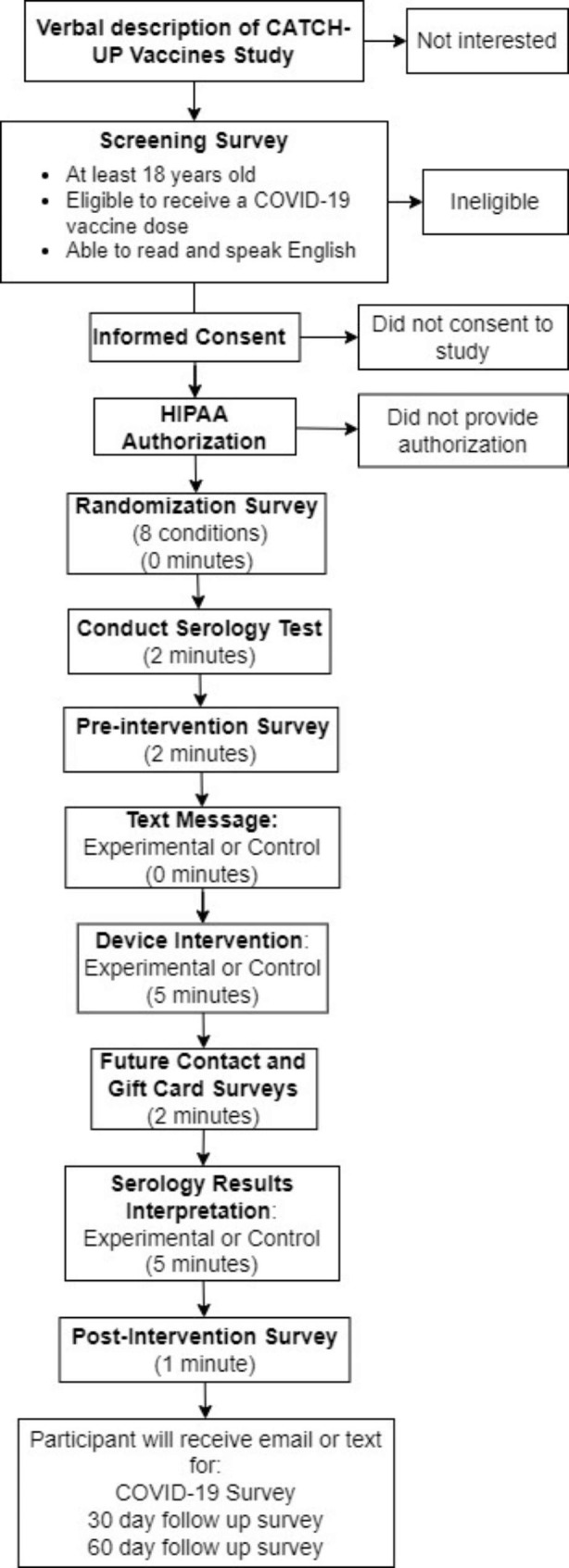
Flowchart of study activities for the randomized controlled trial

During events, we recruit participants through flyers, emails, and direct contact. Participants are eligible for a $20 gift card at the completion of the study, which is given to participants on-site or mailed. If determined to be eligible, we ask participants to provide informed consent and Health Insurance Portability and Accountability Act of 1996 (HIPAA) authorization. The informed consent includes optional consent for future contact for other research studies, sharing ZIP Code (and no other identifiable information) with the NIH RADx-UP Coordination and Data Collection Center, and a follow-up interview for evaluation purposes. Once consent and HIPAA are obtained, participants complete a pre-intervention survey to collect demographic information and reasons to/not to receive a COVID-19 vaccine (Fig. 2).

### Randomization and treatment allocation

Once the pre-intervention survey is complete, participants are randomized through REDCap to a 2 × 2 × 2 fully crossed factorial design, creating eight conditions to evaluate the contribution of each intervention component (Table 1). Study personnel are not masked to the intervention assignment.

### Intervention factors

Interventions address three primary areas to improve vaccination uptake (Table 2). The intervention period of the study is expected to take 15–30 min. Participants may be randomized to receive all three factors (treatment), all three control factors, or any combination of treatment/control (Tables 1 and 2). All participants have the opportunity to receive point-of-care LFA serology antibody testing using a finger stick, but this is not required for participation. The test takes 15 min to process and will return immunoglobulin M (IgM) and IgG antibody response, allowing a semi-quantitative interpretation ranging from no antibody response to a strong positive antibody response based on darkness of the indicator. While the nature of the LFA is qualitative for the presence of IgG or IgM antibodies, the strength of the response correlates well to recent viral exposure or vaccination. While not used for diagnostic purposes, this semi-quantitative nature does provide the opportunity to encourage individuals that are out-of-date for vaccination to receive a booster shot. Following CDC recommendations [30], the test is not used to assess vaccination status and we recommend all individuals to receive vaccinations irrespective of their test results in order to provide maximal protection. While participants are waiting for the results, the participants receive the interventions.

### Text message

Participants are randomized to either receive (1) a text message encouraging COVID-19 vaccination (treatment) or (2) no text message (control). The text language differs depending on whether vaccines are available at the event. If vaccines are available: “[First name], think about getting your COVID-19 vaccine at the event today. All doses are available.” If vaccines are not available: “[First name], think about getting your COVID-19 vaccine today. Find free vaccines at vaccines.gov.”

### Tailored messaging

**Fig. 3:**
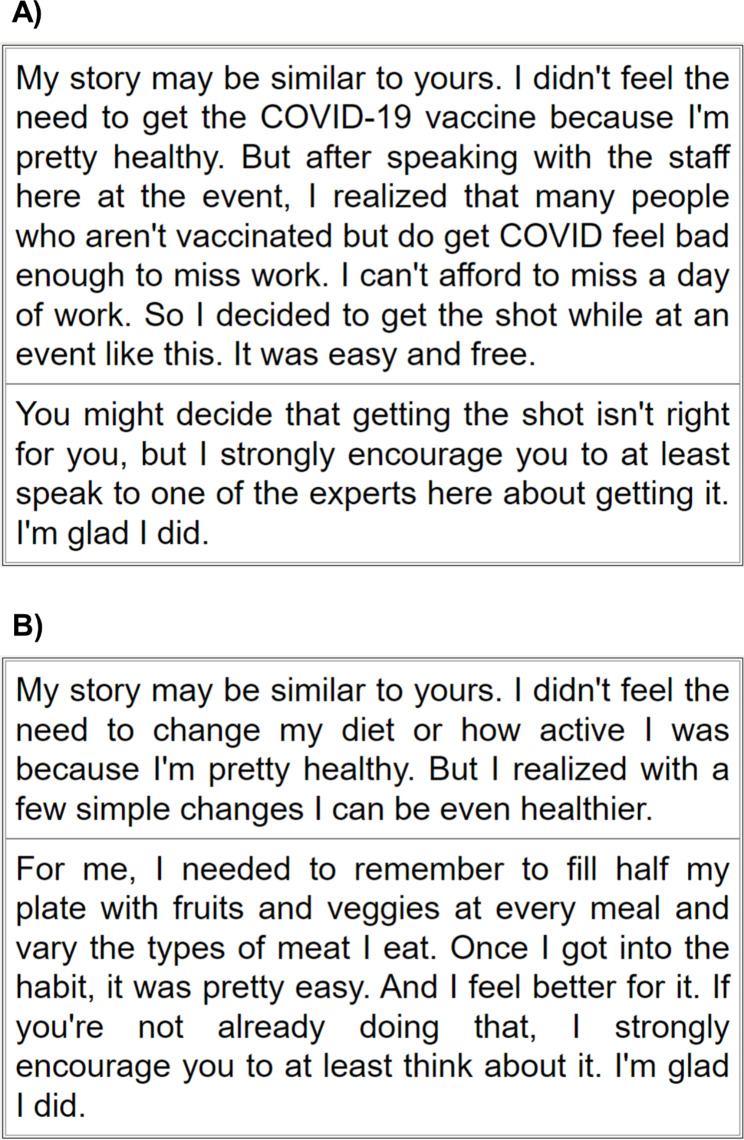
Example tailored messaging personal story for participants randomized to receive (**a**) COVID-19 vaccine educational intervention or (**b**) control wellness message

Participants are randomized to receive (1) educational messaging about COVID-19 vaccines (treatment) or (2) attention control educational message not related to COVID-19 (control). These messages are delivered on a tablet through REDCap and are tailored to vaccine-related concerns that participants report in the pre-intervention survey. Vaccine-specific educational messages use reputable COVID-19 resources from the National Institutes of Health [27]. The attention control message focuses on general health and wellness from the Shape Your Future website [31] and does not address the COVID-19 vaccine (Fig. 3). While we initially planned to format these messages with engaging images (e.g., photographs and cartoons), initial testing identified challenges in bandwidth availability for the larger file sizes in both rural and urban areas. Thus, the final versions are text-based.

### Motivational interviewing

Participants are randomized to receive feedback on their antibody tests by one of these methods: (1) receive interpretation of their antibody results and participate in a motivational interview encouraging vaccine uptake following the 5 A’s strategy (Ask, Advise, Assess, Assist, Arrange) using a semi-structured format (treatment) or (2) interpretation of antibody test results and encouragement of vaccine uptake (as per standard practice) without further prompting by the investigators about reasons to not receive a vaccine (control). The treatment group receives tailored messaging based on concerns reported in the pre-intervention survey that are identical to the messages reviewed in the tailored messaging intervention (if assigned to the treatment group for that intervention). During pilot intervention events, personnel make notes regarding any deviations from the semi-structured script or assigned treatment arm (e.g., some participants may decline to discuss the vaccine) to improve this factor at future events.

Example text interpreting the antibody tests results for a participant with low IgG antibodies, no IgM antibodies, and no history of a COVID-19 vaccine: “Your test results are similar to [show image like theirs]. This image [show high IgG image] is what we would expect to see if you had either had a recent infection with COVID-19 or were up-to-date on COVID-19 vaccines. The faint red line (next to IgG on the participant’s test) means that your antibodies are low because of not being exposed to COVID-19 recently and not being vaccinated. This means that your body is not as prepared to fight a COVID-19 infection as it could be.” Those randomized to the intervention group are further asked if they would like to discuss the vaccine (Ask), discuss their concerns using evidence-based vaccine messaging (as in the Tailored Messaging component) (Advise), ask if they are ready to receive a vaccine that day (Assess), provide guidance to access a vaccine (Assist), inform participant about future follow-up surveys that will also include information on how to access vaccines (Arrange). If participants decline to participate in any step of the 5 A’s, we end the intervention, thank the participant for their time, and encourage vaccine uptake.

## Assessments

### Pre-Intervention

All participants complete a screening form and pre-intervention survey prior to randomization (Table 3). The screening form collects age and vaccine history (manufacturer, number of doses, dates received). The pre-intervention survey requests demographics (race, ethnicity, sex, occupation, county of residence), likelihood of receiving an approved COVID-19 vaccine or booster (if fully vaccinated with the primary series), and reasons to and not to get a vaccine.

### Post-intervention

Once the interventions are complete, the participants complete a brief exit survey that repeats the questions about intention to receive a COVID-19 vaccine or booster and reasons to and not to get a vaccine. The survey is re-sent to participants through text message at 30 and 60 days after the intervention. In addition, we request participants self-report whether they received a vaccine since the intervention. We send up to three daily reminders to the participant to complete the 30- and 60-day follow-up surveys. After the intervention is complete, participants are also sent a survey required by NIH to collect the RADx-UP Common Data Elements through text message or email, with follow-up daily for up to three days after the initial survey invitation is sent.

To evaluate the intervention, we contact up to 60 participants who have agreed to future contact to participate in an interview to help the personnel determine what did and did not work well during the intervention. The interview lasts 30 min to one-hour.

At all events, key personnel conduct a field observation of how the events flow, including external factors such as weather or logistical problems, and where challenges to implementing the study or recruiting participants occurred. At the conclusion of all events, we will conduct evaluation focus groups with event site staff to determine what did and did not work during the event.

**Table 3:** Study tasks and measures across timepoints

Task	Activities/Measures	Enrollment	Pre-Intervention	Intervention	Post Intervention	30 days	60 days
Enrollment	Screening	X					
	Informed Consent/HIPAA	X					
	Allocation		X				
Interventions	Process Improvement			X			
	Barrier Elicitation and Reduction			X			
	Teachable Moment Messaging			X			
Demographics	Race, ethnicity, age, sex, gender, employment status, geography (ZIP code, county, state), contact information for text message		X				
Assessments	COVID-19 vaccine history: Manufacturer, number of vaccines, date of most recent vaccine		X			X	X
	Vaccine status post-intervention: Receipt of a vaccine since participation, receipt of a vaccine at study event					X	X
	Intent to receive a COVID-19 vaccine: Likelihood of getting a COVID-19 vaccine, likelihood of getting a booster shot, reasons to and not to get a vaccine		X		X	X	X

### Statistical analysis

All analyses are intent-to-treat. Outcome measures are assessed at baseline, 30 days, and 60 days follow-up. At the outset, we will examine the frequency distributions of all variables. We will compare the baseline characteristics to assess whether randomization distributed covariates evenly. We will determine whether there is differential dropout and consider developing probability-of-completion weights to obtain unbiased estimates of treatment effect.

The primary analysis will assess the effects of each intervention component in terms of its association with the primary outcome (intent to receive a COVID-19 vaccine immediately after the interventions are completed), comparing each intervention individually with the outcome. The primary outcome question is ordinal, with responses ranging from “very likely” to “definitely not”. We will analyze this variable as continuous and calculate the difference in score pre v. post intervention. For binary association analysis, we will use chi-square tests or Fisher’s exact tests (when sample size is small) for categorical variables and two-sample t-tests or Wilcoxon rank sum tests, whichever is more appropriate, for continuous variables. If there are no significant differences in covariates by intervention group, we will use one-way analysis of variance (ANOVA). However, if there are significant differences in covariates across intervention groups, we will use multiple linear regression to compare the difference in intent to receive a COVID-19 vaccine by intervention group accounting for potential confounders. We will evaluate the assumptions for linear regression and transform data as appropriate. Missing values are excluded from the analysis. All analyses will use an alpha of 0.05 and are conducted in SAS v. 9.4 (Cary, NC).

### Sample size and power calculations

Power calculations are based on our primary analysis, which is to assess the effect of each intervention component on increased intention to receive a COVID-19 vaccine immediately after the intervention comparing a single intervention group to the control. We expect to recruit 400 participants during the study. A sample size of 80 (control group plus one intervention group) achieves above 85% power to detect an R-Squared of 0.1 attributed to one independent variable (binary indicator of control or intervention group) using an F-Test with a significance level (alpha) of 0.05. The variables tested are adjusted for an additional 1 to 5 independent variables with an R-Squared of 0.1. Since we expect to recruit 400 participants during the study, we expect to have more than 80 participants for the combination of control group and any intervention group. The power calculations were conducted in PASS 11.

### Trial status and modifications

We are initially conducting a pilot intervention that will include randomization to receive a text message and tailored messaging, with all participants receiving the control arm of the motivational interviewing factor. During these pilot events, staff are assigned to observe and take notes on the interaction to help develop the treatment arm of the motivational interviewing factor. If enrollment is lower than anticipated at individual community events, we will work with PHIO to identify additional community sites and pair the intervention with other activities with large attendance (e.g., health fair, staff training day, etc.). All changes will be submitted to the institutional review board (IRB) for review and approval prior to implementation.

Recruitment is ongoing and we plan to complete recruitment for the Aim 2 intervention in June 2023, with 60-day follow-up completed by August 31, 2023. We will disseminate results of the study through scientific presentations, including through the RADx-UP initiative and through published manuscripts. De-identified data on required Common Data Elements will be deposited into the NIH RADx-UP Data Hub throughout the study period.

## Discussion

We expect to obtain information on interventions that are likely to increase uptake of the COVID-19 vaccines from both focus group data and discussions with key informants in the partnering communities. We anticipate that information obtained from pilot interventions will provide insights in improving interventions to increase COVID-19 vaccine uptake in underserved communities. This study will focus on designing and testing a multilevel process of improving COVID-19 vaccine uptake, but it will not have the resources to assess the underlying details of individual care steps closely. For example, this study will not provide a detailed examination of the processes underlying clinical decision support, academic detailing, etc., as exploration of these outcomes would require resources that go beyond the scope of the present study. However, community experience in vaccine uptake will be explored through robust qualitative inquiries to elucidate cultural values, preferences, and needs related to COVID-19 vaccine uptake that are specific to underserved populations in Oklahoma. Our study purposefully incorporates community-based collaborations that can be replicated in other settings (e.g., diversifying the skills of existing personnel and building upon exiting infrastructure) to ensure generalizability.

There is always some uncertainty in how the landscape of vaccination for COVID-19 will change over time, including resources, personnel, infrastructure, leadership, and eligibility for vaccination. However, our team has shown in past studies [32–35] that it is possible to minimize the impact of time-bound uncertainties on the success of community-engaged health improvement programs by establishing durable relationships and engagement with stakeholders and ongoing adaptation to their needs. Incentivizing community participation in research is always a challenge. However, we have carefully aligned this study with the internal goals and ongoing strategic healthcare programs of our community partners, so leadership buy-in has already been accomplished. The study team will work closely with our community partners to find ways to provide additional support that may help community partners continue participation if they experience unforeseen challenges.

Compared to the US overall, Oklahoma has lower COVID-19 vaccine uptake, particularly in rural areas of the state. Thus, it is important to identify community-driven and community-delivered interventions to address vaccine hesitancy. The MOST framework provides an innovative and timely opportunity to efficiently evaluate multiple educational interventions in a single study and is also being applied in COVID-19 testing interventions in underserved communities [12].

## Data Availability

Not applicable as this manuscript is a protocol paper and data collection is ongoing. As part of the RADx-UP program, de-identified data on Common Data Elements will be deposited into the NIH RADx-UP Data Hub. Additional trial data will be available to researchers upon reasonable request and execution of data sharing agreements.

## Abbreviations

MOST: multiphase optimization strategy
CATCH-UP: Community - engaged Approaches to Testing in Community and Healthcare Settings for Underserved Populations
PHIO: Public Health Institute of Oklahoma
OUHSC: University of Oklahoma Health Sciences Center
RADx-UP: Rapid Acceleration of Diagnostics – Underserved Populations
PCR: Polymerase chain reaction
LFA: Lateral flow assay
NIH: National Institutes of Health
REDCap: Research Electronic Data Capture
CDC: Centers for Disease Control and Prevention
HIPAA: Health Insurance Portability and Accountability Act of 1996
IgG: Immunoglobulin G
IgM: Immunoglobulin M
ANOVA: Analysis of variance
IRB: Institutional Review Board
